# Single-cell transcriptomics reveals age-resistant maintenance of cell identities, stem cell compartments and differentiation trajectories in long-lived naked mole-rats skin

**DOI:** 10.18632/aging.204054

**Published:** 2022-05-04

**Authors:** Aleksandra Savina, Thierry Jaffredo, Frederic Saldmann, Chris G. Faulkes, Philippe Moguelet, Christine Leroy, Delphine Del Marmol, Patrice Codogno, Lucy Foucher, Antoine Zalc, Mélanie Viltard, Gérard Friedlander, Selim Aractingi, Romain H. Fontaine

**Affiliations:** 1 Université Paris Cité, CNRS, INSERM, Institut Cochin, Paris, France; 2 Institut de Biologie Paris Seine (IBPS), Laboratoire de Biologie du Développement, Sorbonne Université, CNRS, INSERM, Paris, France; 3 Fondation pour la Recherche en Physiologie, Brussels, Belgium; 4 Queen Mary University of London, School of Biological and Chemical Sciences, London, United Kingdom; 5 Service d'Anatomie et Cytologie Pathologiques, Hôpital Tenon, Assistance Publique-Hôpitaux de Paris, Paris, France; 6 Université Paris Cité, CNRS, INSERM, Institut Necker-Enfants Malades, Paris, France; 7 Université de Namur ASBL, Namur, Belgium; 8 Ecole Nationale Vétérinaire d'Alfort, Centre de Recherche Biomédicale, Maisons-Alfort, France; 9 Service de Dermatologie, Hôpital Cochin, Assistance Publique-Hôpitaux de Paris, France

**Keywords:** naked mole-rat, skin, stem cells, wound healing, aging

## Abstract

Naked mole-rats (NMR) are subterranean rodents characterized by an unusual longevity coupled with an unexplained resistance to aging. In the present study, we performed extensive *in situ* analysis and single-cell RNA-sequencing comparing young and older animals. At variance with other species, NMR exhibited a striking stability of skin compartments and cell types, which remained stable over time without aging-associated changes. Remarkably, the number of stem cells was constant throughout aging. We found three classical cellular states defining a unique keratinocyte differentiation trajectory that were not altered after pseudo-temporal reconstruction. Epidermal gene expression did not change with aging either. Langerhans cell clusters were conserved, and only a higher basal stem cell expression of *Igfbp3* was found in aged animals. In accordance, NMR skin healing closure was similar in young and older animals. Altogether, these results indicate that NMR skin is characterized by peculiar genetic and cellular features, different from those previously demonstrated for mice and humans. The remarkable stability of the aging NMR skin transcriptome likely reflects unaltered homeostasis and resilience.

## INTRODUCTION

Skin acts as an essential barrier and protects organism from external threats, preventing fluid loss, stabilizing body temperature, and relaying sensory information to the brain. Maintaining skin homeostasis is essential, as alterations in skin functions can cause various deleterious conditions ranging from fluid loss to more severe diseases such as infections or UV-induced cancers. Because the skin is frequently wounded, maintenance of an adequate protection requests rapid and finely tuned cutaneous repair steps. In addition to epidermal stem cells committed to the formation, differentiation and regeneration of the epidermis, the skin harbors an elaborate network of immune cells including subsets of T cells but also a substantial component of myeloid-derived cells such as dendritic cells and Langerhans cells (LC). All these specialized immune cells within the skin provides the first line of defense, protecting the body against infections [1, 2].

Constantly exposed to both internal and environmental stresses such as UV radiation or air pollutants, the skin ages and undergo profound changes in its appearance and functions. Indeed, aged skin undergoes gradual structural and functional degeneration, leading to thinning of epidermal and dermal layers, loss of elasticity, wrinkling and dryness. These changes are responsible for delayed wounding, more frequent infections, pruritus, enhanced allergen/irritant penetrations with variable degree of dermatitis and eventually carcinogenesis. In rodents and humans, these phenomena have been partially attributed to loss or lineage skewing of keratinocytes stem cells and immune cells, and/or the regulation of their niches, altering normal homeostasis and tissue repair [3–7].

Aging is often described as an inevitable and irreversible process. However, one species seems to defy the natural laws of aging: the naked mole-rat (NMR, *Heterocephalus glaber*). The NMR are small poikilothermic and hairless rodents native to East Africa, where they live underground in eusocial colonies. These mouse-sized rodents live almost five times longer than expected on the basis of body size, with a maximum lifespan exceeding 37 years in captivity and up to 17 years in their natural habitat [8–11] Despite being the longest-lived rodent, NMR do not show any increase in age-specific hazard of mortality in defiance of Gompertz’s law [12] and all of the classical signs of aging such as decreased fertility, muscle atrophy, bone loss, changes in body composition or metabolism seem to be mostly absent in these animals [13]*.* Moreover, the incidence of age-related diseases such as metabolic disorders, neurodegenerative diseases and even cancers are absent or extremely low in NMR [14, 15]. The NMR genome has been sequenced in 2011 revealing a unique genetic profile and molecular adaptations correlated with all the characteristics described above: resistance to cancer, poikilothermia, absence of pelage, resistance to low levels of oxygen, as well as impaired vision, taste and circadian rhythms. 93% of the NMR genome has a synteny with the human, rat or mouse genome [16]. Thereby, the NMR, as an example of successful slow-aging mammal, emerges as a highly attractive organism for the study of skin aging. Investigation of the NMR skin has revealed unique adaptations to thermoregulation and their extreme environmental challenges. The skin morphology and histology of the NMR have been studied, however NMR skin biology during aging is still unexplored [17–20].

We used single-cell RNA-sequencing (scRNA-seq), to obtain the unbiased molecular RNA profile of the NMR epidermal cell populations. By profiling 10,000+ cells from skin epidermis in young and older NMR, we found that epidermal compartments and cell populations, especially the stem cells pool, remained unaffected despite aging. *Igfbp3*, expressed by keratinocyte stem cells and known to play a major role during epidermal homeostasis, was found upregulated in older animals, contrary to what is observed in other species. In addition, functional skin healing experiments revealed that the wound closure rate was strictly comparable between young and older animals.

## RESULTS

### Histomorphology of young versus middle-aged NMR skin

**Figure 1:**
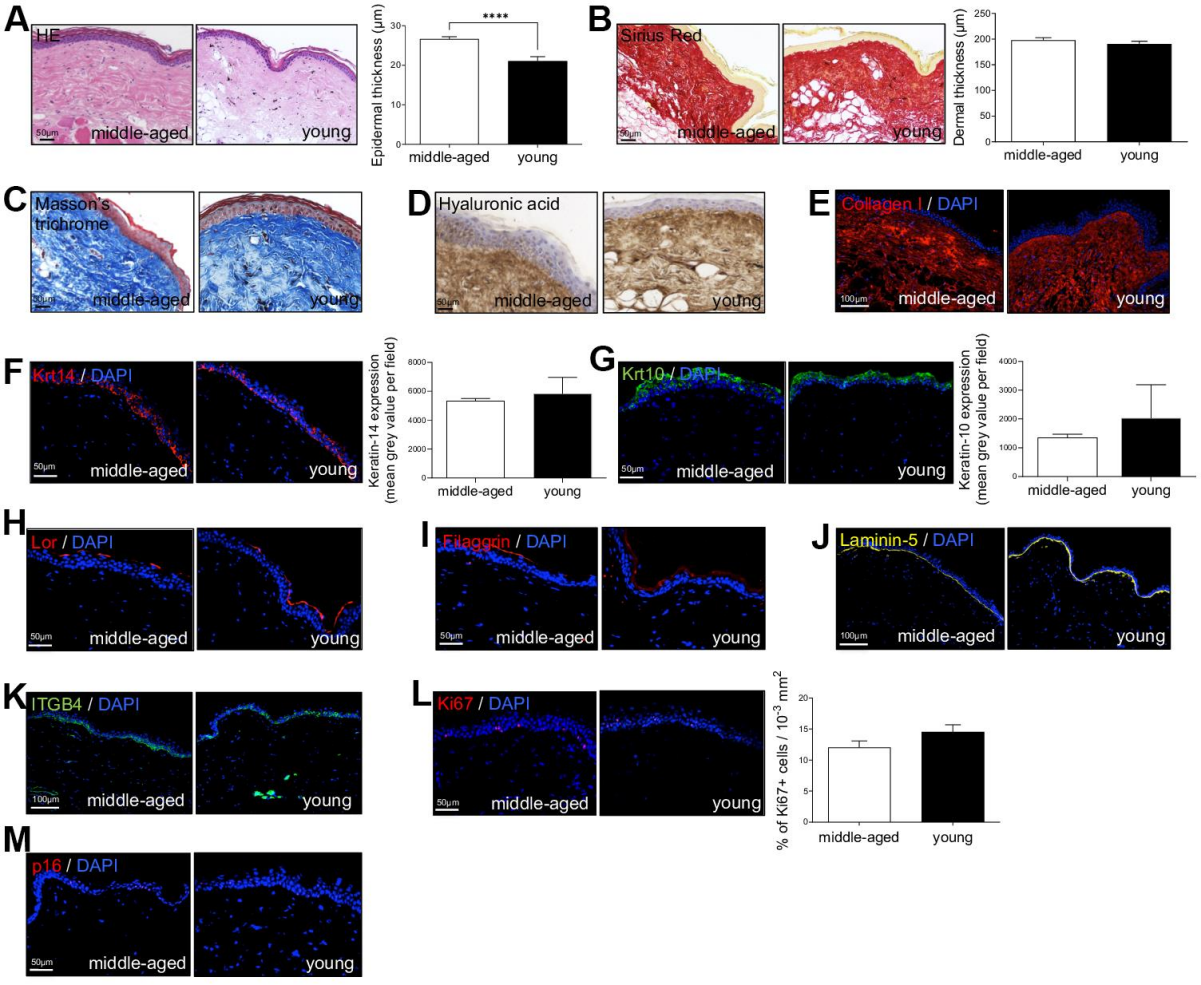
**Naked mole-rat (NMR) skin histomorphology.** (**A**–**C**) HE, Sirius Red and Masson’s Trichrome staining photomicrographs on sections of middle-aged and young NMR back skin. Histograms in A and B show the epidermal and dermal thickness (μm) of middle-aged and young NMR back skin. Scale bar = 50 or 100 μm. Bars: SEM. *represents differences between the age groups. ****p≤0.0001 in Student’s t-test. n=4 animals per group. (**D**–**M**) Hyaluronic acid, Collagen I, Keratin-14 (Krt14), Keratin-10 (Krt10), Loricrin (Lor), Filaggrin, Laminin-5, ITGB4, Ki67and p16 staining photomicrographs on sections of middle-aged and young NMR back skin. Cells were stained with either Alexa 546 or Alexa 488 fluorochromes. For hyaluronic acid, cells were stained with 3,3’-diaminobenzidine. Negative controls were performed in parallel with the samples substituting the primary antibody with the equivalent isotype. Histograms show the quantification of Krt14 and Krt10 expressions and Ki67+ cells per surface in both age group. Scale bar = 50 or 100 μm. Bars: SEM. A Mann–Whitney U test was used for Keratin-14 / Keratin-10 quantification and a Student’s t-test was used for Ki67+ cells quantification. n=4 animals per group.

First, to investigate NMR skin structure and organization changes associated with cutaneous aging, dorsal skin of young (Y, mean age: 1.1 years) and middle-aged (MA, mean age: 11.3 years) animals were collected and evaluated by immunohistochemistry. HE and Sirius Red staining were used to measure epidermal and dermal thickness of NMR skin (Figure 1A, 1B). In human and mouse, skin thickness decreases with age. Surprisingly, NMR epidermis was significantly thicker in older animals (histogram in Figure 1A), and the global histomorphological structure was unchanged during aging. This increased epidermal thickness was confirmed in older 20-25 years NMR tissue samples (only available for histology analysis, Supplementary Figure 1A–1C). The number of epidermal cells (Supplementary Figure 1D) and epidermal layers (Supplementary Figure 1E) were significantly higher in the MA group. More precisely, cell counting per layer revealed an additional cellular layer (“layer 6”) in older animals (Supplementary Figure 1F). No effect of age was observed on the dermal thickness between Y and MA animals (histogram in Figure 1B). Masson’s trichrome staining for collagenous connective tissue fibers showed no difference between the 2 groups just like hyaluronic acid, a linear polysaccharide of the extracellular matrix contributing to contact inhibition and cell proliferation [21] or collagen I (found in all dermal layers; Figure 1C–1E). Epidermal layers immunostaining using anti-Keratin-14 (Krt14, basal layer marker), Keratin-10 (Krt10, suprabasal layer marker), Loricrin (Lor, a major protein of the cell corneous envelope), Filaggrin (intermediate filament-associated protein of the outer granular layer of the epidermis), Laminin-5 (dermal-epidermal basement membrane protein), Integrin-beta4 (ITGB4, basal surface marker juxtaposed to the dermal-epidermal basement membrane) antibodies showed no difference between Y and MA animals (Figure 1F–1K). It has been discovered that epidermal proliferation declined with age especially in humans [4]. Interestingly, in NMR, Ki67 index for cell proliferation revealed no significant difference between Y and MA NMR (Figure 1L). p16 (or CDKN2A), one of the most frequently used markers of senescence and an important hallmark of aging, was slightly detected in NMR skin but again, no differences were observed between the 2 age groups (Figure 1M) whereas this marker accumulates in an age-dependent manner in mammalian skin [22]. Thus, the results suggest that NMR skin aging is delayed.

### scRNA-seq of NMR epidermis defines 3 cellular states for NMR keratinocytes

The skin epidermis is constantly rejuvenated by keratinocyte stem cells found in the basal layer that are capable of both proliferation and differentiation. But aging skin experiences a progressive decline in homeostatic and regenerative capacities, potentially attributed to degenerative changes in these stem cells and/or their niches [3–7]. Using single-cell RNAseq, we analyzed the cellular composition of the NMR epidermis to investigate the potential link with their slower rate of skin aging.

**Figure 2:**
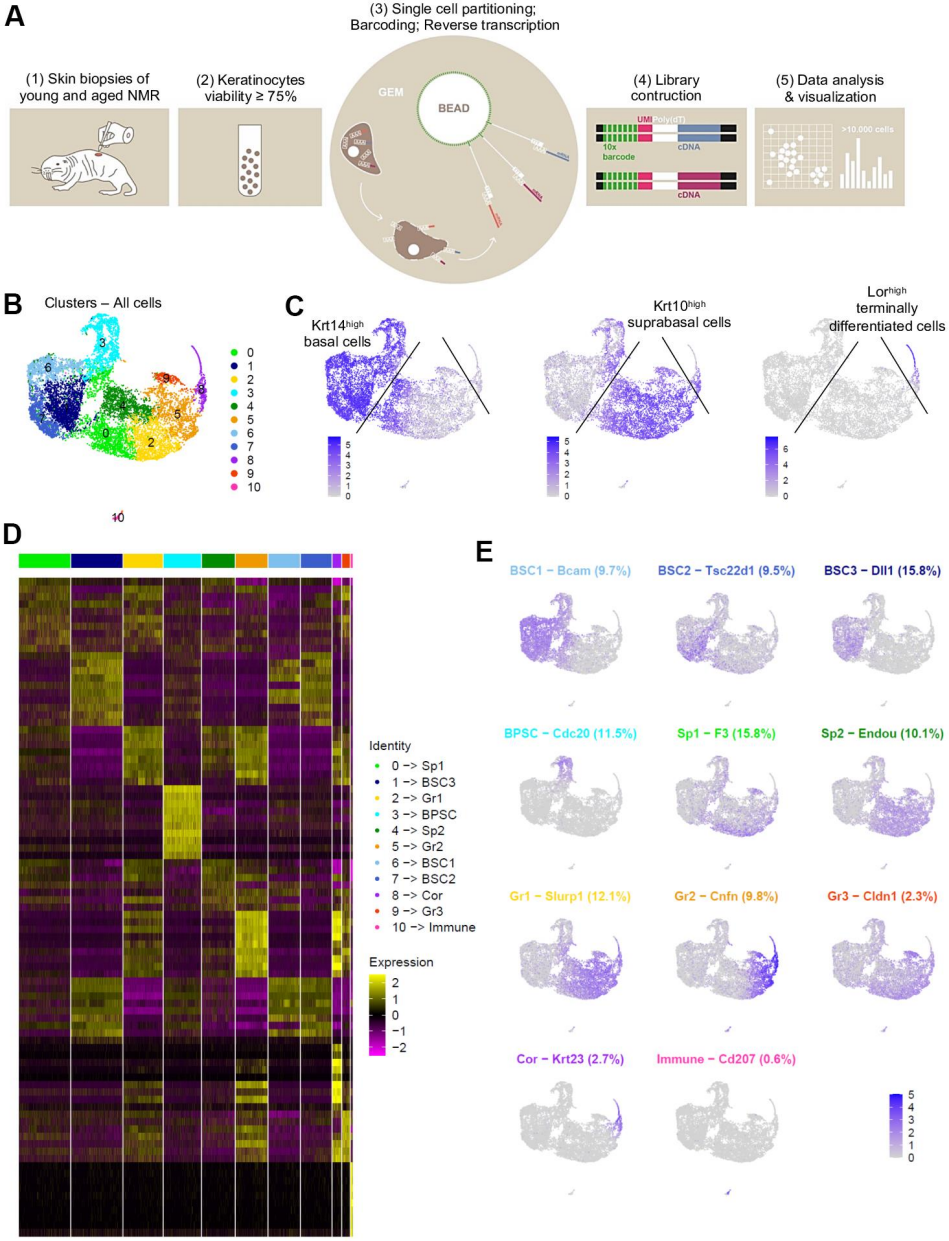
**Cell populations characterization of NMR epidermis using single-cell RNA-sequencing (scRNA-seq).** (**A**) Overview of the experimental workflow. (**B**) Global epidermal cell transcriptomes (n=10,232 cells) visualized with Uniform Manifold Approximation and Projection (UMAP), colored according to unsupervised clustering to better compare different cell subtypes. 11 different cell clusters were found, numbered from 0 to 10. (**C**) Expression of the 3 main marker genes during epidermal differentiation (*Krt14, Krt10, Lor*) projected onto UMAP. Cells were sub-divided into 3 main groups according to the differential epidermal cellular states: basal (*Krt14^high^*), suprabasal/intermediate (*Krt10^high^*) and corneous (*Lor^+^*) layers. Cells with the highest expression level are colored dark blue. (**D**) Heatmap of differentially expressed genes. For each cluster, the most differentially expressed genes and their relative expression levels in all sequenced epidermal cells are shown. Cells are represented in columns, and genes in rows. 3 selected genes for each cluster were color-coded and annotated on the right. The 10 clusters were identified as: basal and stem cells BSC1-2-3 for clusters 6-7-1; basal proliferating and stem cells BPSC for cluster 3; spinous layer cells Sp1 and Sp2 for clusters 0-4; granular layer cells Gr1-2-3 for clusters 2-5-9 respectively; corneous layer cells Cor for cluster 8; immune for immune cells cluster 10. (**E**) Expression levels for each cell are color-coded and overlaid onto UMAP plot for one selected cluster-specific gene. Cells with the highest expression level are colored dark blue. % of cells in each cluster is annotated.

After a short enzymatic epidermis-dermis digestion and separation, epidermal cells were mechanically dissociated and directly loaded into the chromium controller (10x Genomics) without any FACS-sorting to prevent cells from stress (Figure 2A). All the samples were processed in parallel to minimize batch effects. Quality control (# UMI ≥ 2000, # genes ≥ 700, % MT ≤ 25%, number of cells) was made with FastQC, Cellranger and Seurat v3 pipeline (Supplementary Figure 2A–2D). Unsupervised clustering revealed that one sample from the MA group had a very distinctive pattern after Principal Component Analysis (PCA). UMAP (Uniform Manifold Approximation and Projection) visualization for all individual samples is presented in Supplementary Figure 2E. This sample (2V) was removed, keeping for the rest of our study 3 samples in the Y NMR group and 2 samples in the MA NMR group (see materials and methods). In total, we obtained 10,232 cells from 5 NMR epidermis for downstream analyses. Based on gene expression profiles and UMAP to visualize cell clusters, we found 11 cellular populations (numbered from 0 to 10, Figure 2B). To identify marker genes of each cluster, we used differential expression testing, with a Wilcoxon rank sum test. These marker genes were used to establish the cell identity of each cluster, together with additional markers identified through literature searching for cell types typically found in the human or mouse skin and mining existing single-cell transcriptomic skin data (Supplementary Data File 1). We found 2 different populations: keratinocytes representing 99,4% (10,171 cells) in clusters 0 to 9, and immune cells in cluster 10 representing 0.6% of epidermal cells (61 cells). This immune-isolated cluster 10 contained a majority of *Cd207*^+^/*Ctss*^+^/*Mfge8^+^* LC [23, 24] (Supplementary Data File 1). We did not find cells expressing melanocytes genes, which is consistent with previous reports showing that melanocytes were only detected in the NMR dermis [17].

**Figure 3:**
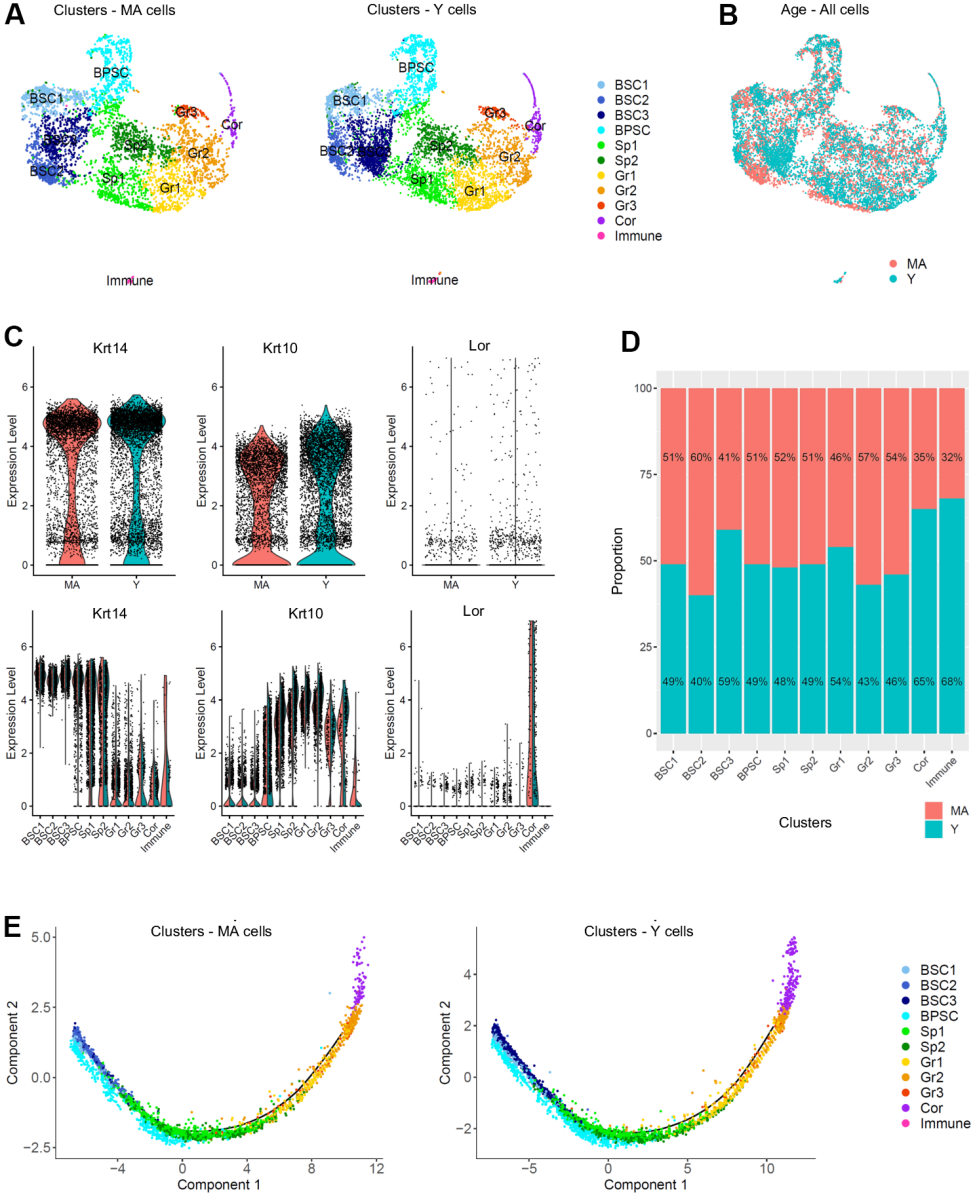
**Single cell populations and trajectories analysis of young versus middle-aged NMR epidermal cells reveals no difference between the 2 age groups.** (**A**) Uniform Manifold Approximation and Projection (UMAP), colored according to unsupervised clustering from middle-aged (MA; n=4,218 cells) and young (Y; n=6,014 cells) NMR epidermal cells. Cell populations from both MA and Y NMR were unchanged as the same 11 clusters were detected. (**B**) Cells from both MA (red) and Y (blue) NMR were jointly projected on the same UMAP plot, showing an overlap of the 2 age groups tested. (**C**) Violin plots of *Krt14, Krt10* and *Lor* marker genes expressed by MA (red) and Y (blue) epidermal cells (upper panel) and segregated by cluster (lower panel). No detectable changes in their expression were found. (**D**) Bar graph representing the relative proportion of epidermal cells in each cluster between MA (red) and Y (blue) animals. There was no significant difference in epidermal cell proportion between the 2 age groups using Chi^2^ statistic test (pval ≤ 0,05). (**E**) Unsupervised differentiation trajectories constructed with M3Drop and Monocle v2.10.1 for MA and Y keratinocytes. Immune cells’ cluster 10 has been excluded to focus on epidermal keratinocytes. The results showed a unique trajectory with no branches in both samples and the same repartition of cluster among the trajectory. BSC = basal and stem cells; BPSC = basal proliferating and stem cells; Sp = Spinous layer cells; Gr = granular layer cells; Cor = corneous layer cells.

Cytokeratin expression can globally define the differentiation status of keratinocytes: *Krt5* and *Krt14* are expressed in undifferentiated basal stem and proliferative cells, while *Krt1* and *Krt10* are found in differentiating suprabasal cells and *Lor* occupies 80% of the epidermal corneous envelope [25–27]. Thus, using these well-defined marker genes, we were able to confirm that in NMR, the keratinocyte population also separates into 3 main cellular states. Four clusters (clusters 1-3-6-7) expressed high levels of *Krt14* (*Krt14^high^*) and *Krt5* and were classified as basal cells. Clusters 0-2-4-5-9 expressed higher levels of *Krt10* (*Krt10^high^*) and *Krt1* and were categorized as suprabasal or intermediate cells. The last keratinocyte cluster (cluster 8) was the only one to express very high levels of Lor (*Lor^high^*) and was considered as a cluster of terminally differentiated keratinized cells of the corneous layer (Figure 2C). The same results were confirmed with violin plots of these marker genes (Supplementary Figure 3A) and t-distributed Stochastic Neighbor Embedding (t-SNE) visualization (Supplementary Figure 3B, 3C). The most differentially expressed marker genes for each cluster were represented using heatmap visualization (Figure 2D) and 3 specific genes for each cluster were highlighted and color-coded to match cluster color. For each cluster, the most representative gene was projected onto the UMAP plot (Figure 2E).

In order to confirm these 3 cellular states in NMR keratinocytes, we identified other well-known skin layer genes in each cluster. In *Krt14^high^* basal cells clusters, cells from clusters 1-6-7 expressed specific basal layer genes such as *Tp73, Itga6, Itgb1, Cxcl14 and Smoc2* [27–30]. Cells from cluster 1 expressed the Notch ligand *Dll1,* a marker of stem cells found in the basal layer in human and mouse skin [28, 31], *Igfbp3*, a gene exclusively expressed in the basal layer’s proliferative keratinocytes in normal skin [4, 28, 32, 33], and *Socs3*, a gene known to promote maintenance of epidermal homeostasis [28]. Of note, *Dll1* and *Igfbp3* were also expressed in the two other basal clusters 6 and 7. Cluster 6 cells expressed *Bcam*, *Ascl2* and *Rarres2*, 3 genes found in the basal layer of the epidermis and known to stimulate keratinization [34, 35]. *Tsc22d1*, *Tnc*, *S100* family members (such as *S100a4* and *S100p*) and *Ccl2* expressed in Cluster 7 cells were reported to be restricted to basal/follicular stem cells and progenitors with high cell turnover notably during wound healing [28, 33, 36]. *Krt14^high^* cluster 3 cells specifically expressed classical epidermal cell cycle regulators such as *Cdc20, Ube2C and Cdkn3* [33, 37, 38] (Figure 2E)*.* Concerning *Krt10^high^* suprabasal cells clusters 0-2-4-5-9, we found that *Notch* receptor gene was expressed in clusters 5 and 9 cells, while its target gene *Hes1* was found in cluster 0 and 4. It has been demonstrated that *Notch* activation induces terminal epidermal differentiation through *Hes1* which is expressed in the spinous layer. The expression of another Notch ligand, *Jag2* was restricted to basal layer’s clusters 1-6-7 cells corroborating previous findings. *Jag1* on the other hand was only found in suprabasal cluster 9 cells [31]. In addition to *Hes1*, cluster 0 cells also expressed *F3* and *Ehf* reported to be located in suprabasal layers of the skin playing an essential role in keratinocyte differentiation [28, 39]. Cluster 4 analysis revealed the expression of *Endou*, *Ly6D* and *Mt4*, 3 genes previously associated with keratinocyte differentiation in the spinous layer of the skin [28, 30]. Of note, *Mt4*, reported to specify a transitory stage from basal to spinous layer [28, 36], was expressed in cells from cluster 0, 4 and 2. Cluster 2 cells strongly showed classical suprabasal gene expression such as *Krt1, Krt10* and *Slurp1*, known to be a marker of late differentiation state, predominantly expressed in the granular layer of the skin [40]. In cluster 5 cells, *Cnfn*, *Ly6g6c and Clic3* expression matched with a granular layer phenotype [28]. The last suprabasal cluster defined here, cluster 9, was also consistent with genes expressed by differentiating keratinocytes in the granular layer of human and mouse skins such as *Cldn1, SerpinB12* and *Lypd3* [28]. In addition, both clusters 5 and 9 cells revealed the expression of several strong makers such as *Hspb1* and *Grhl1* suggesting the possible granular keratinocytes phenotype of these two cluster cells [28, 38] (Figure 2E)*.* Finally, in addition to *Lor*, cluster 8 cells expressed *Klk5/7*, *Krt23*, *Cdsn* or *Lce5a* strongly suggesting these cells were terminally differentiated keratinocytes from the corneous layer [28] (Figure 2E, Supplementary Data File 1). We were then able to label these keratinocytes clusters. Undifferentiated cells from Clusters 1-6-7 were named basal and stem cells BSC3, BSC1 and BSC2 respectively. Cluster 3 was named basal proliferating and stem cells BPSC. Cluster 0 and 4 were defined as spinous layer cells Sp1 and Sp2, cluster 2-5-9 as granular layer cells Gr1-2-3 respectively. At last, cluster 8 was labeled as corneous layer cells Cor (Figure 2D, 2E).

The single cell transcriptomic analysis of NMR keratinocyte unraveled, for the first time in NMR, an epidermal differentiation process that follows 3 classical cellular states, from basal layer’s proliferative stem cells and/or progenitors (BSC and PPSC clusters) to suprabasal (Sp1-2 and Gr1-2-3 clusters) and terminally differentiated cells (Cor cluster). Within each of these three cell population, each cluster could represent a specialized subpopulation.

### Cell populations and trajectories analysis of NMR keratinocytes define metaclusters but reveal no age-related differences

To study any transcriptional changes of the NMR epidermis during aging, we compared Y and MA NMR epidermis. Three Y NMR of 1.1 years old (n=6,014 cells) and two MA NMR of 11.3 years-old (n=4,218 cells) were analyzed. Y and MA cells seemed to be equally spread across clusters using UMAP visualization (Figure 3A, 3B). This observation was confirmed using t-SNE visualization (Supplementary Figure 4A). In addition, the clusters’ cellular states, defined by the expression of *Krt14*, *Krt10* and *Lor* marker genes and visualized with violin plots, were identical between the two age groups (Figure 3C). The proportion of cell types in each cluster after normalization was not statistically different between the 2 conditions (Chi^2^ statistic test pval ≤ 0,05; Figure 3D). To identify differentially expressed genes between Y and MA animals, we then compared the expression of genes between all cells from Y samples and all cells from MA samples. No gene was found differentially expressed using the following thresholds: p-value below 0.05 and average log fold-change (logFc) above 1. This result was surprising because it is now well known that aging induces the differential regulation of multiple growth-promoting genes. For example, 1237 genes were found differentially expressed in the skin of mice at 9 months, 15 months, and 24 months [41].

As keratinocytes differentiate, they undergo a process of transcriptional reconfiguration. Using Monocle 2, we reconstructed the single cell trajectory of Y and MA NMR keratinocytes to mimic their biological differentiation process. Of note, cluster 10 cells corresponding to epidermal immune cells was excluded from all Monocle analysis to focus only on the keratinocytes’ differentiation program. Colored by cluster, we found the same linear trajectory for each age condition, with no branches, suggesting an absence of different cellular decision or alternative expression program between Y and MA keratinocytes (Figure 3E). For both Y and MA NMR, the distribution of keratinocytes along their process of cellular differentiation showed that, as expected, *Krt14^high^* clusters BSC1-2-3 were condensed at the root of the trajectory process, with cluster BPSC is spreading out more from this root showing their early engagement in the differentiation process. Then, intermediate *Krt10^high^* clusters Sp1-2 and Gr1-2-3 cells moved towards the right of the trajectory, as they were more committed in the differentiation process (no cells were observable in the root). And finally, *Lor^high^* Cor cluster cells were distributed at the very end of the trajectory, for terminally differentiated keratinocytes [42] (Figure 3E). These results were also represented by Monocle states (state or “segment of the tree”, Supplementary Figure 4B) and for each cluster (on separate plots) for merged samples or Y and MA samples separately, to see precisely where each cluster was located (Supplementary Figure 4C).

**Figure 4:**
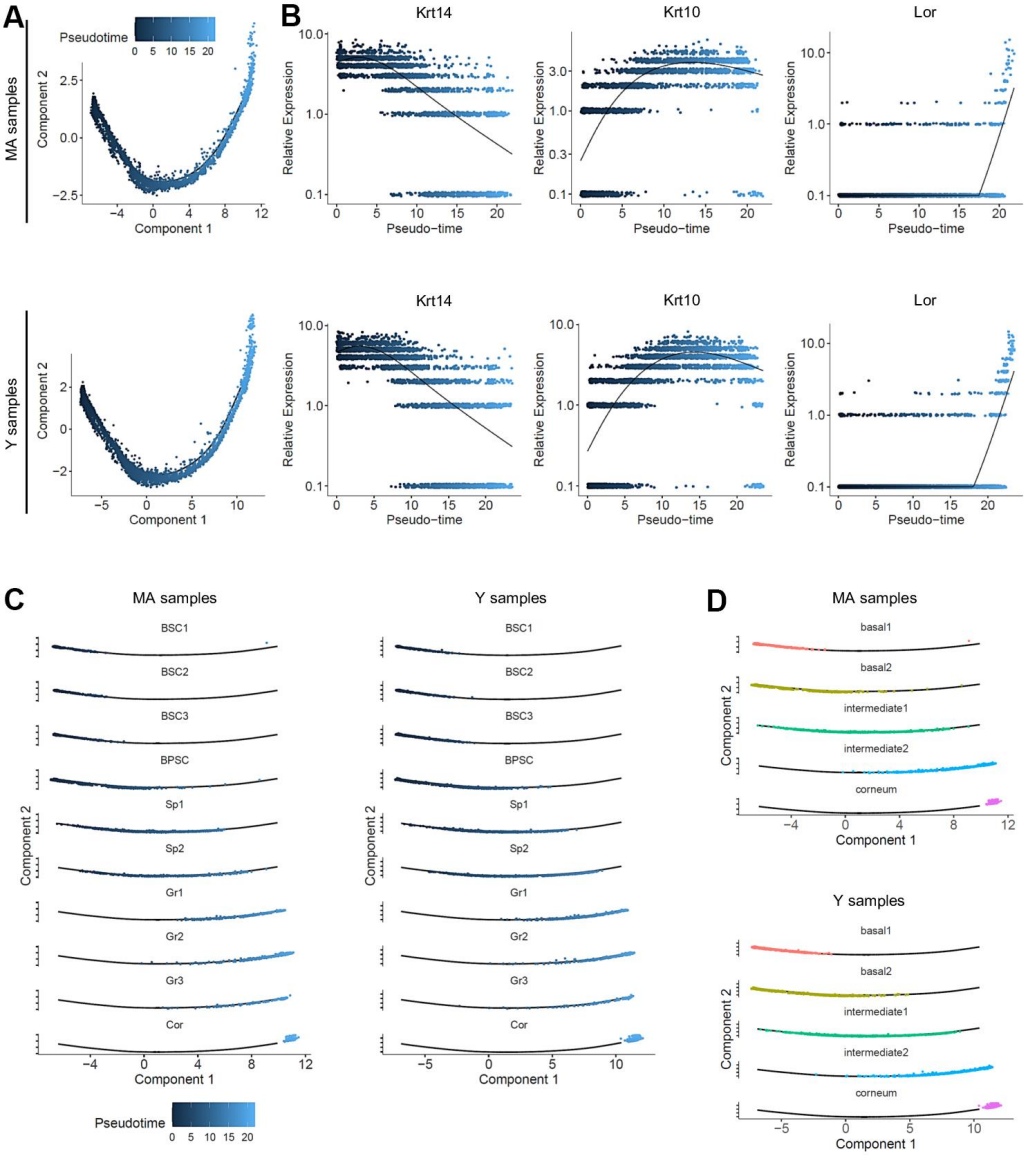
**Pseudotime analysis and metacluster identification in young versus middle-aged NMR keratinocytes.** (**A**) Colored pseudotemporal ordering of keratinocytes for MA (middle-aged) and Y (young) NMR separately. No significant differences were observed between the 2 groups. (**B**) Relative expression of *Krt14*, *Krt10* and *Lor* markers plotted along the pseudotime axis for each condition. The same relative expression profile was found between MA and Y NMR, validating the 3 cellular states for NMR keratinocytes and the no age-related impact of aging in the differentiation process. (**C**) Pseudotime estimation showing epidermal progression along the differentiation process with individualized cluster visualization, for each condition. Five metaclusters were defined according to their differentiation engagement: basal 1, basal 2, intermediate 1, intermediate 2 and corneum. (**D**) Unsupervised differentiation trajectories for MA and Y animals. The same repartition of metacluster among the trajectory was observed. BSC = basal and stem cells; BPSC = basal proliferating and stem cells; Sp = Spinous layer cells; Gr = granular layer cells; Cor = corneous layer cells.

Colored by pseudotime, the cells were also visualized according to a blue gradient which becomes lighter as cells move away from the root of the trajectory, reflecting their maturation state. No significant differences on pseudotime estimation were found between Y and MA NMR (Figure 4A). To validate our 3 cellular states in NMR keratinocytes, the 3 selected well-known gene markers of epidermal differentiation *Krt14, Krt10* and *Lor* [42] were plotted according to their mean expression levels (black line) along the pseudotime axis (Figure 4B). No changes between the 2 age groups were observed suggesting an absence of age-related effect on keratinocyte differentiation in NMR (Figure 4B). To assign a more precise cellular state of differentiation to the 10 clusters, we chose to individualize them on separate plots (Figure 4C). Thus, combined with data relative to specific gene expression obtained in Figure 2, pseudotime reconstruction allowed us to order the 10 clusters precisely and to partition them into 5 metaclusters (i.e group of clusters) according to their differentiation engagement: i) basal 1 containing BSC1-2-3 clusters; ii) basal 2 for BPSC cluster; iii) intermediate 1 containing Sp1-2 clusters; iv) intermediate 2 aggregating Gr1-2-3 clusters and finally v) terminally differentiated cells for the Cor cluster. The expression of *Krt14, Krt10 and Lor* marker genes visualized with violin plots confirmed the cellular states of these newly defined metaclusters (Supplementary Figure 5A). The repartition of cell types between the Y and MA animals after normalization was not statistically significant in each metacluster (Chi^2^ statistic test pval ≤ 0,05; Supplementary Figure 5B). Distributions of the frequency of Y and MA cells as a function of the pseudotime on its scale were also plotted. Wilcoxon test revealed that Y and MA NMR keratinocytes distributions were similar using the pseudotime estimation (p-value = 0.1673). According to pseudotime segments, no different gene markers were found between the segments (Supplementary Figure 5C). Then, we observed the most differentially expressed genes and their relative expression levels in all sequenced epidermal cells on a heatmap (Supplementary Figure 5D). Similar to the previous analysis, five metaclusters of genes could be clearly identified, each with a different region of activity during the differentiation process. Unsupervised differentiation trajectories for Y and MA animals showed identical repartition of these metaclusters among the trajectories (Figure 4D).

To conclude, we identified 11 different clusters defining epidermal cells from Y and MA NMR and a single path cell trajectory. Using pseudotemporal ordering, we were able to identify 5 metaclusters. Basal metaclusters containing keratinocytes stem cells and proliferative progenitors seemed to contribute to all the other cellular states to promote epidermal differentiation, up to the upper corneous layer. Taken all the results together, comparing young and older NMR keratinocytes, we observed a well-maintained skin tissue and cellular identities throughout the years, contrary to what has been described in mouse or human [4, 27].

### Metaclusters characterization and enrichment analysis for specific functions of NMR keratinocytes

As basal keratinocytes transit through different cellular states until terminal differentiation, we examined the dynamics of the genetic profiles used to construct the trajectories using Monocle. We identified 6 gene modules following similar kinetic gene changes along pseudotime. The heatmap profiles were identical, suggesting that the same gene groups at the same expression level were orchestrating the differentiation process in both Y and MA NMR keratinocytes, independently of aging (Supplementary Figure 6A, left panel). We then investigated whether specific functions could be assigned to those 6 groups using Gene Ontology (GO). The most significant GO terms found in each activated gene group for both ages comforted their implication in their commitment : early activated cluster 5 for basal layer state, cluster 1 for cell cycle still in the early pseudo-timeline (note the sharp transition between these 2 states), mid-time activated cluster 6 for cell differentiation, cluster 3 at the end of differentiation program for an ultimate transition, cluster 2 strictly circumscribe at the end of pseudotime differentiation process and finally cluster 4 harbouring a biphasic expression pattern with functions involved at various times of the differentiation process supported by different genes (Supplementary Figure 6A, right panel).

Next, another GO analysis was performed on the different epidermal metaclusters defined above. We used the most representative markers of each metacluster and described a repartition of function for each of them (Supplementary Figure 6B; Supplementary Data File 2). Basal 1 metacluster (BSC1-2-3 clusters) was characterized by developmental and cell adhesion processes relative to their basal stem cell/progenitor phenotypes. Indeed, those cells were likely prone to establish cell junction to the extracellular matrix (ECM) and to commit to a global developmental process of tissue formation. As expected, basal 2 metacluster (BPSC cluster) was well defined by a wide range of cell cycle functions relative to their mitotically active progenitor cell type. Intermediate 1 (Sp1-2 clusters) and intermediate 2 (Gr1-2-3 clusters) metaclusters were characterized by cell differentiation and developmental processes confirming these clusters as a population of differentiated keratinocytes of the spinous and granular layers respectively. Lastly, corneum metacluster (Cor cluster) was represented by active lipid metabolism as well as cell death regulation and reactive oxygen species (ROS) reaction terms (Supplementary Figure 6B; Supplementary Data File 2). The characteristics of metaclusters were well maintained in both age groups.

Furthermore, due to the link between ROS and aging, we performed an enrichment analysis for our metaclusters for aging and ROS-related genes. Surprisingly, none of the aging-related gene analyzed were found enriched in any of our metaclusters. However, 4 ROS-related genes were significantly enriched in corneum metacluster (Supplementary Figure 6C and Supplementary Data File 3). Indeed, *Prdx6, P4hb, Sod1* and *Prdx2* were enriched in the Corneum metacluster (Supplementary Figure 6C and Supplementary Data File 3; pval 1,01^e-02^). In NMR, the abundantly expressed antioxidant enzyme *Sod1* activity was 1.35-fold higher as compared to age-matched mice but age did not have an effect on its activity in both species. Peroxiredoxins, especially *Prdx2 and Prdx5,* were shown to be expressed at lower levels in NMR livers, which could result in increased levels of ROS and suggest that the long-lived NMR can thrive despite elevated oxidative stress [43]. Of note, *Prdx 2* and *5* were also enriched in the immune cluster. Those immune cells expressed high levels of transcripts related to regulation of immune system process and response to stimulus, showing that their main function is to be involved in resistance to skin pathogens. Those terms were also found at a lower level in the corneum metacluster where immunity meets external antigens, and in the basal 1 metacluster where keratinocytes have key roles in immune defense (Supplementary Figure 6D and Supplementary Data File 3).

### *Igfbp3* is overexpressed in older NMR skin

**Figure 5:**
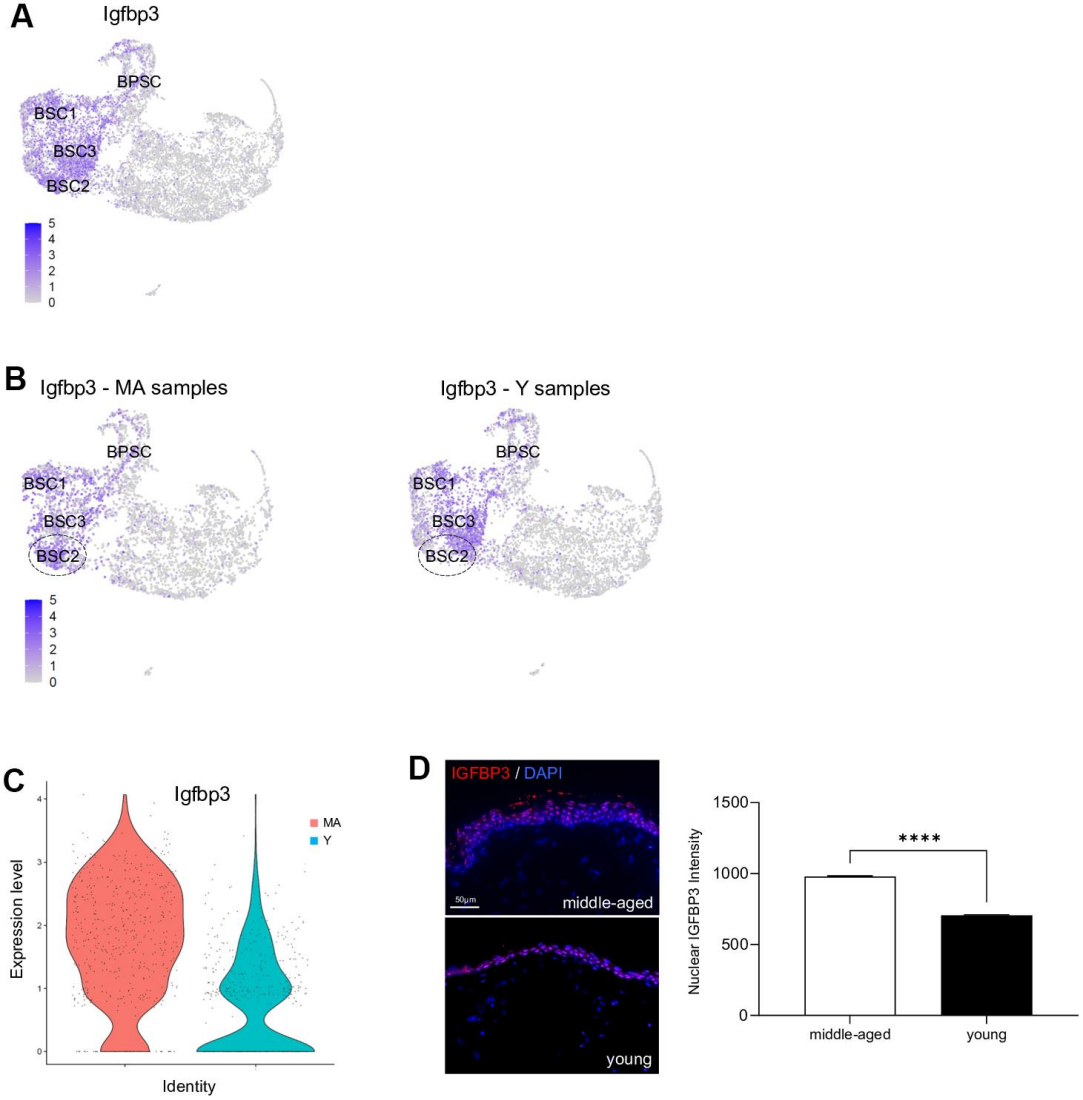
***Igfbp3* expression in young versus middle-aged NMR keratinocytes.** (**A**) Expression levels for *Igfbp3* gene onto Uniform Manifold Approximation and Projection (UMAP). Note that its expression is restricted to basal stem and proliferative cell clusters BSC1-2-3 and BPSC. (**B**) Expression levels for *Igfbp3* gene onto UMAP for MA (middle-aged) and Y (young) NMR. The dotted circle delimits BSC2 cluster. (**C**) Violin plots of *Igfbp3* genes expressed by MA (red) and Y (blue) epidermal cells. (**D**) IGFBP3 staining photomicrographs on sections of MA and Y NMR back skin. Cells were stained with Alexa 546 fluorochromes. Negative controls were performed in parallel with the samples substituting the primary antibody with the equivalent isotype. Scale bar = 50 μm. Histogram showing the quantification of nuclear IGFBP3 expression in both age group. Bars: SEM. *represents differences between the age groups. ****p≤0.0001 in Student’s t-test. n=4 animals per group.

Previous studies have demonstrated that cutaneous gene expression levels change with age, but NMR cutaneous gene expression showed a remarkable stability with age (see above). However, as subtle differences might exist, we performed a more in-depth analysis between Y and MA animals within each cluster independently. Using a p-value below 0.05 and an average logFc above 1, only 2 differentially expressed genes in BSC2 cluster (*ENSHGLG00000002542/Rps2* and *Igfbp3*) were overexpressed in MA NMR (Supplementary Data File 4). *Rps2* gene encodes a ribosomal protein that is a component of the 40S subunit, with unknown role in skin homeostasis. Interestingly, *Igf/Igfbp* signaling plays an important role in aging processes and mediates epidermal proliferation and skin peripheral immunity. *Igfbp3,* one of six IGF binding proteins, was found located in the basal/germinative layer of the epidermis and modulates epidermal homeostasis. Here, using UMAP visualization, we retrieved its expression in basal stem and proliferative clusters BSC1-2-3 and BPSC (Figure 5A). In mice and humans, *Igfbp3* transcript is significantly reduced with increasing age [4, 28, 32, 33]. In NMR, *Igfbp3* expression was almost 4 time higher in the BSC2 cluster of older animals as compared to the younger ones (Figure 5B, 5C and Supplementary Data File 4). Confirming our scRNA-seq data, IGFBP3 nuclear expression assessed by immunohistochemistry was increased in older animals (Figure 5D). Moreover, 5 differentially expressed genes in immune cluster (*S100a9*, *ENSHGLG00000004445/S100a8*, *Krt17*, *S100p*, and *Krt5*) were overexpressed in older animals. Interestingly, no differentially expressed genes typical of LC were found, suggesting the stability of these cells over time in the NMR (Supplementary Data File 4).

### Wound healing is not affected by age in NMR skin

**Figure 6:**
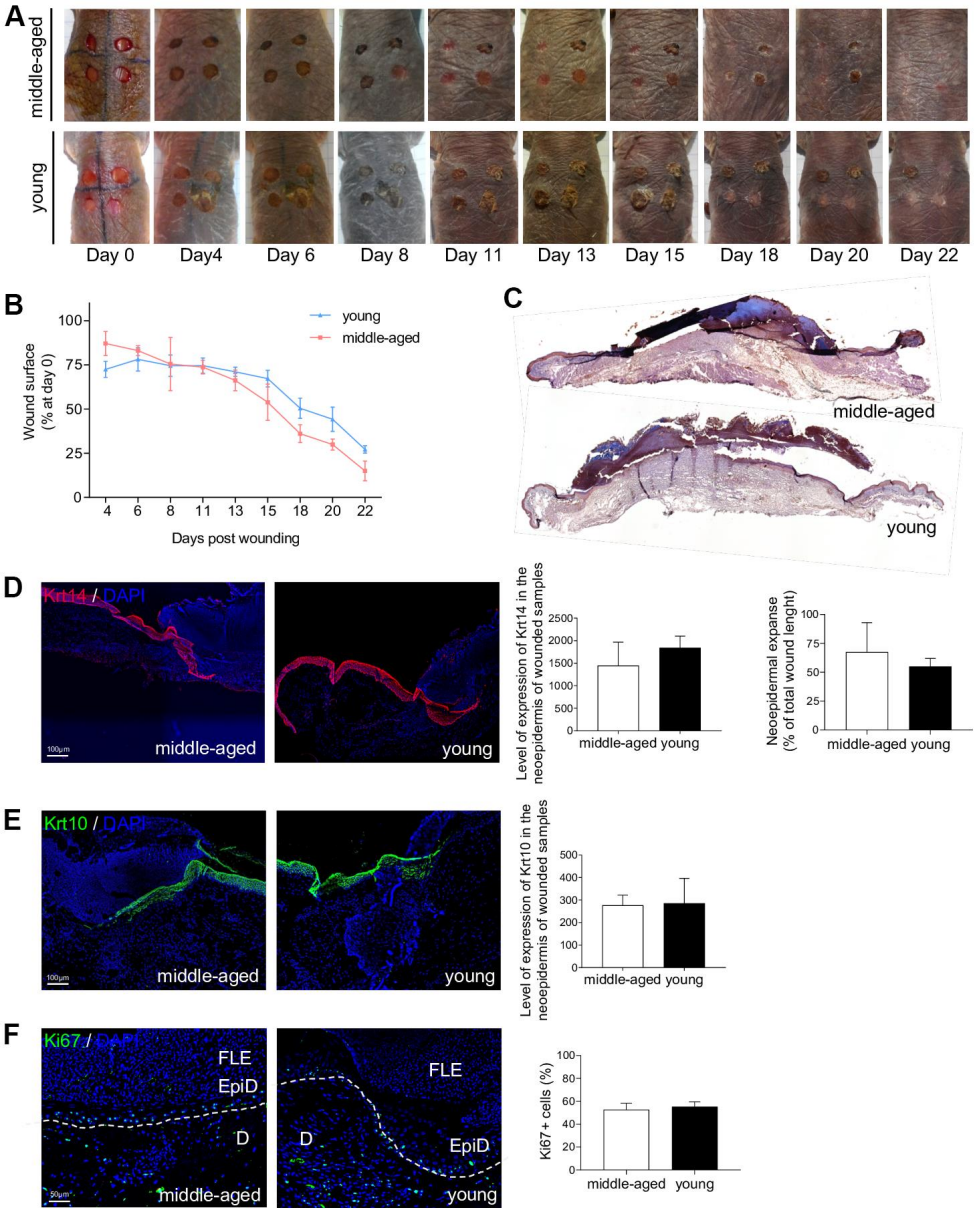
**NMR wound healing.** (**A**) Photomicrographs of back skin wounds for middle-aged and young animals from day 0 to day 22 post wounding. (**B**) Wound closure rate (% of wound surface at day 0) from day 4 to day 22 post wounding. No statistical difference was found the 2 groups using Student’s t-test. n=4 animals per group. (**C**) HE staining photomicrographs on sections of middle-aged and young NMR back skin 7 days post wounding. (**D**, **E**) Krt14 and Krt10 staining photomicrographs on sections of middle-aged and young NMR neoepidermal tongues in the wound at day 7. Histograms show the level of expression of Keratin-14 and Keratin-10 in the neoepidermis of wounded samples (mean grey value per field) and the percentage of re-epithelialization was calculated according to the formula: [length of the extending epidermal tongues]/[length of the wound] x100%. (**F**) Ki67 staining photomicrographs on sections of middle-aged and young NMR neoepidermal tongues in the wound at day 7. Histogram show the percentage of Ki67+ cells in epidermal wound edges representing the growth fraction of an epidermal tongue cell population. Legends: epidermal wound edges (EpiD), dermis (**D**), fibrino-leukocyte exudate (FLE). In (**D**–**F**), cells were stained with either Alexa 546 or Alexa 488 fluorochromes. Negative controls were performed in parallel with the samples substituting the primary antibody with the equivalent isotype. Scale bar = 50 or 100 μm. Dash lines represent the limit between epidermis and dermis. Bars: SEM. Student’s t-test were used. n=2 animals per group.

The ability of the skin to heal quickly and completely is a major function that characterizes the skin status. Epidermal stem cells, and their crucial role in the cutaneous healing process, have been extensively studied in mice and humans [33, 44] but never in NMR. Stem cell dysfunction is a hallmark of aging and is associated with impaired wound healing in older rodents and humans [5, 7, 45]. The delayed skin aging in the NMR prompted us to investigate the wound healing process in young and older NMR. Punch biopsy devices were used to create surgical wounds on the backs of Y and MA NMR and the wound closure rate was measured (percentage of wound closure at day 0) for the first time in these animals. Unlike normal mice that possess a healing time course of approximately 5 days, NMR appeared to heal at a slower rate of 22 days but with less scarring (Figure 6A, 6B). In humans and mice, wound closure occurs more rapidly in younger than older individuals [5, 7, 45]*.* Our results showed that in NMR, despite an age difference of 11 years, the wound closure rate of MA animals followed the same kinetic as the one of Y animals (Figure 6B). Next, we investigated re-epithelialization at day 7 post-wounding as an essential component of wound healing and used as a defining parameter of its success (Supplementary Figure 7A). A well-organized neo-epidermis of the same thickness between Y and MA was measured (Figure 6C and Supplementary Figure 7B). In all individuals, the same levels of expression of basal Krt14 and differentiated Krt10 cell markers were found and the neo-epidermal tongue expanse was substantially identical between the 2 groups (Figure 6D, 6E). The epidermal tongue proliferation rate measured using Ki67 immunostaining remained stable between Y and MA animals (Figure 6F).

Metaclusters enrichment analysis for wound healing related genes revealed that 3 genes were significantly enriched from a list of 74 genes in Basal 1 metacluster: *Cav1, Dst, Dsp* (Supplementary Figure 6C and Supplementary Data File 3; pval 5,86^e-03^). Mutations in the mouse *dystonin* (*Dst*) and *desmoplakin* (*Dsp*) genes have previously been shown to cause skin fragility [46, 47]. *Cav1* has been found in undifferentiated epidermal basal layer keratinocytes and considered by others as a potential epidermal stem cell marker [48]. Moreover, some studies have proven that disruption of *Cav1* expression can lead to age-related changes in skin and aberrant wound healing [49]. Interestingly, *Igfbp-3* can be internalized independent of IGFs into the cell through caveolae-mediated endocytosis.

## DISCUSSION

Age-related epidermal changes can be caused by multiple elements leading to: reduction of keratinocyte stem cell compartment, alteration of cellular proliferation, thinning of the epidermis, slowing of the keratinocytes turnover, flattening of the dermal-epidermal junction or the regulation of the stem cell niche. The results of our investigation clearly showed that NMR skin ages differently than rodent or human skin [3, 4, 27, 38, 50, 51]. First, skin structure and organization appear to be well maintained between young and older NMR. Second, using scRNA-seq, we found that their epidermal cells displayed 2 main populations, immune cells (1 cluster) and keratinocytes, subdivided into 10 clusters. The immune cells cluster was easily distinguished from the other cell clusters with a characteristic signature (*Cd207, Ctss, Mfge8*) corresponding mainly to LC. By ordering the 10 keratinocytes clusters, we found a classical differentiation process including basal stem cells and proliferative keratinocytes, followed by spinous then granular layer cells and finally terminally differentiated cornified cells. The results of the present study suggest that NMR epidermal homeostasis remain unaltered for the first 11 years of their life and for some parameters up to 25 years old. Indeed, epidermal cell identities, cellular states and differentiation trajectory were highly similar in older animals. Recently, single cell analysis was successfully used to detect differences between young and adult mice epidermis, showing that young epidermal cells progenitors appeared more homogenous compared with adult cells progenitors. Moreover, these authors found only one differentiation trajectory in young mice but 2 in older mice, while others have found up to 3 distinct trajectories in adult mice interfollicular epidermal cells [28, 33, 36]. Using also single-cell transcriptomic data, The Tabula Muris Consortium assessed cell-type-specific age-related changes in *Mus musculus tissues*. In skin, these authors found that the relative abundance of keratinocyte stem cells decreased significantly with age with at least 20 differentially modulated genes in skin computed by age (3, 18, 21 and 24 months) [50]. Another recent article confirmed that human epidermal thickness decreased constantly over time, with thinner skin already observed in MA individuals (35-48 years old). In addition, unlike the NMR, thousands of differentially expressed genes were identified in the human aging skin. Further analysis of these differentially expressed genes highlighted that MA individuals were closer to old individuals at the transcriptomic level, indicating the early onset of human skin aging [51]. These data could indicate that NMR are better protected against skin aging through the mainstay of gene expression, contrary to what has been shown in mice and humans [41, 51].

Performing a deeper analysis within each cluster individually, we found 2 genes overexpressed in basal stem cells of older animals and 5 genes overexpressed in immune cells of older animals. In basal stem cells, *Igfbp3* was four times higher in older animals*, while* in mice and humans, there is a large decrease of this gene during skin aging [4]. This analysis also revealed that the level of LC appeared unaffected through aging in the NMR. While, in mouse and human epidermal aging, a decrease in the number of LC has been proven; their numbers and maturation state are reduced in aged mice starting at 12 months of age [6]. In addition, in NMR cutaneous immune cells, there were overexpressed genes belonging to the *S100* and *Keratin intermediate filament* families, known to be regulators of inflammation and immunity in the skin. To date, only few studies using scRNA-seq profiling have focused their attention on NMR immunity, but no data on skin immune cells were available. First, authors found that NMR immune system was characterized by a high myeloid to lymphoid cell ratio as compared to mice but only healthy young NMR were used [52]. More recently, NMR aged 3 and 11 years were used as compared to mice aged 3 and 12 months. They discovered that NMR owned an additional pair of cervical thymi and that they had no thymic involution up to 11 years of age, suggesting that they displayed a delayed immunosenescence [53]. Thus, we hypothesize that the maintenance of cellular compartments in the older NMR, especially the stem cell pool through high *Igfbp3* expression, coupled with an increase skin immunity, could explain their skin slower rate of aging. In addition, a recent study suggests that NMR skin could be immune to senescent cells build-up through a unique mechanism: senescent cell death, an immune-independent apoptosis of senescent cells. This mechanism could reduce the chance of senescent cell accumulation, a hallmark of skin aging [54].

Age-related skin changes have a deleterious impact on water loss, body temperature, and wound healing, making the skin more susceptible to long-standing wounds. Surgical excision wounds performed on the back of the NMR, showed an impressive maintenance of healing kinetics in older animals. A well-organized neo-epidermis with a clear neo-epidermal tongue expanse was found regardless of the age of the animals. Once again, these results are in contrast with what has been shown in mouse and human [5, 7, 45]. For example, wound closure has been proven to occur more rapidly in young mice (6 months) than in middle-aged (15 months) or aged mice (26 months; [45]). This important finding demonstrates that NMR skin remains protected against one of the worst consequences of skin aging.

At this point, several other elements could be discussed. First, the MA animals were the oldest animals currently available in our laboratory. However, 11 year-old NMR are considered old in the wild where only few animals live beyond 10 years and most die at age 1-2 years, but are considered middle-aged in captivity where mortality increases well beyond 25 years [8–10]. Second, it is important to note that while MA NMR skin structure was found to be similar to that of young animals, the thickness of the epidermis appeared paradoxically increased at an older age, as a consequence of increased number of epidermal layers. Marked thickness of the corneous layer in the adult NMR has previously been described [18] and lack of fur has also been described to be compensated by a thicker epidermis [17]. The reason for such an increase remains unknown. It could result from a reaction to the higher cumulative skin friction in their tunnel habitats for older animals. It could also be due to the overexpression of *Igfbp3* in the basal/germinative layer of the epidermis in older animals, leading to a modulation of the early stages of epidermis differentiation. Third, it should also be noted that we focused our study on skin epidermal keratinocytes although it has been demonstrated that human dermal fibroblasts undergo a partial loss of their cellular identity during aging [27]. Finally, alterations in hair follicle stem cell niche -that occur in mice aging- was not possible to address in this hairless rodent [38].

To conclude, NMR live more than 37 years, while they should only live for 6 years, based on their size. Therefore, the maintenance of their epidermal stem cell and LC compartments -possibly in relation with increased *Igfbp3* expression and markers of immunity- appear to contribute to their remarkable longevity and resistance to skin aging. Our data also confirm the relevance of the NMR as a model organism for studying cutaneous biology and disease resistance and provide valuable clues to better understand the cutaneous biology of this animal.

## MATERIALS AND METHODS

### Animals

Young (Y, mean age: 1.1 years) and middle-aged (MA, mean age: 11.3 years) males were obtained from four distinct colonies maintained at the Ecole Nationale Vétérinaire de Maisons-Alfort (EnVA). Animal experiments were performed according to experimental protocols following European Community Council guidelines and approved by the Institutional Animal Care and Use Committee of the Ecole Nationale Vétérinaire de Maisons-Alfort (n°016).

### Histology and immunohistochemistry

MA and Y NMR skin samples were collected under anesthesia by performing 5mm skin punch biopsies on their back skin. The skin was then immerged in formalin for 12 to 24 hours at 4° C, and embedded either in paraffin or OCT compound for sectioning.

Formalin-fixed paraffin-embedded skin sections (5 μm) were stained with Hematoxylin and Eosin, Sirius Red and Masson’s Trichrome using standard procedures. Formalin-fixed paraffin-embedded skin sections or cryo-sections were also labeled with various antibodies (Supplementary Table 1). Briefly, for formalin-fixed paraffin-embedded sections, a prior antigen retrieval was performed by incubating the slides with citrate buffer (pH=6) or EDTA buffer (pH=9) at 98° C for 20 min. Sections were then washed with Tris buffer saline (TBS) (137mM NaCl and 20mM Tris) / Tween 20 (TBST) 0.1% and blocked for 2 hours with TBST/Normal Goat Serum (NGS) 5%. The sections were incubated with the different primary antibodies diluted in blocking buffer overnight at 4° C for intracellular staining, followed by secondary antibody goat anti-mouse Alexa 546 (dilution 1:1000, Life Technologies), or goat anti-rabbit Alexa 488 (dilution 1:1000, Life Technologies) for 45 min at room temperature. Slides were also incubated with 4’,6-diamidino-2-phenylindole (DAPI, Sigma-Aldrich) for 5 min for nuclei staining and mounted using Fluoromount G (Southern Biotech, Birmingham, AL, USA). For hyaluronic acid staining, the slides were incubated 2h with HA-binding protein (at 1,6/100 dilution) with 0.2% bovine serum albumin and 0.02% TritonX-100 in PBS. HA-binding protein staining was achieved with 1/100 strepavidin HRP (Perkin Elmer, Waltham, MA, USA) solution for 30 min at room temperature and 3,3’-diaminobenzidine (Liquid DAB + substrate Chromagen System; Dako, Santa Clara, CA, USA) for color development. Finally, sections were counterstained with hematoxylin (Sigma-Aldrich, St. Louis, MO, USA) and mounted using DPX (HA staining in brown). Negative controls were performed in parallel with the samples substituting the primary antibody with the equivalent isotype.

The sections were visualized under an Olympus BX63F microscope equipped with an Olympus DP73 camera (and pictured with Metamorph software; Olympus, Tokyo, Japan). Using Image J software, epidermal, dermal thickness and number of epidermal layers and cells were measured. Epidermal cells or layers were counted in 10-3mm2 randomized surface squares in the epidermis of each specimen. Quantification was done on various parts of the skin, dissected in a gridded fashion and 3 different sectors were analyzed; for each different sector of the skin, 3 different areas of 10-3mm2 were counted. Similarly, immunolabeled cells were counted and fluorescence intensity was measured with mean gray value per field. For IGFBP3 staining, cell nuclei were automatically segmented and counted using a deep learning-based segmentation algorithm called Cellpose (https://www.cellpose.org/).

### Single-cell RNA-sequencing

Epidermal cells from 3 Y and 3 MA NMR were used. All the samples were collected and processed at the same time. NMR were anesthetized by the inhalation of 4.9% isoflurane delivered at a flow rate of 300 ml min^−1^ in ambient air and ethanol was applied topically to the dorsal skin for 20 seconds. Punch biopsy devices were used to create 5 mm surgical wounds on the backs of the animals. To preserve cell viability, skin pieces were immediately placed in PBS/BSA 3% medium on ice until dissection. The wounds were rinsed with betadine solution. Using a scalpel, the subcutaneous fat was removed from the dermis. The cleaned tissues were placed dermis side down in a sterile petri dish in a trypsin 0.25%/EDTA 1mM solution at 37° C for 1h. The enzymatic digestion was stopped by addition of PBS/FBS 10%. This floating procedure allows the digestion and dermis-epidermis separation by lifting the epidermis up straight above the dermis. Epidermis was then mechanically dissociated and minced in small pieces with razor blades in PBS and then triturated in 25mL, 10mL then 5mL pipets. 3 rounds of washes were performed starting with 100μm filtration, 70μm and 40μm and interspersed with centrifugation cycles at 150g at 4° C for 5 minutes. Finally, the cell pellet was resuspended in PBS/BSA 3% and cell viability was measured using trypan’s blue method on Kova slides. All single cell suspensions had a viability greater than 75%. Single cells then were captured in droplet emulsion using the Chromium Controller (10× Genomics, Pleasanton, CA, USA), and scRNA-seq libraries were constructed according to the 10× Genomics protocol using the Chromium Single-Cell 3′ Gel Bead and Library V2 Kit (10× Genomics, Pleasanton, CA, USA). In brief, cell suspensions were diluted in PBS/FBS 3% to a final concentration of 1 × 10^6^ cells/mL (1,000 cells per μL). Cells were loaded in each channel with a target output of 5,000 cells per sample. All reactions were performed in a C1000 Touch Thermal Cycler (Bio-Rad Laboratories, Hercules, CA, USA) with a 96 Deep Well Reaction Module. Twelve cycles were used for cDNA amplification and sample index PCR. Amplified cDNA and final libraries were evaluated using a Bioanalyzer 2100 (Agilent Technologies, Santa Clara, CA, USA) with a high sensitivity chip. Samples were sequenced on an HiSeq 4000 instrument (Illumina, San Diego, CA, USA).

### scRNA-seq pre-processing

scRNA-seq data analyses were performed by GenoSplice technology (http://www.genosplice.com). Sequencing data quality analysis was performed using FastQC v0.11.2 on 6 NMR epidermal cells samples, 3 from a Y NMR group (samples 1J, 5J, 6J) and 3 from an MA NMR group (samples 2V, 3V, 4V). For read alignment and unique molecular identifiers (UMI) quantification, CellRanger software v3.0.2 was used on Heterocephalus glaber genome HetGla_female_1.0 with default parameters and gene annotation from Ensembl 96. The 6 expression matrices containing the UMI counts were merged, and only the genes with UMI ≥ 1 in at least one cell were kept. The following filters were applied to generate a global matrix used in further analysis: cells with UMI ≥ 2000, number of detected genes ≥ 700, and cells with UMI in mitochondrial genes ≤ 25%. For UMIs normalization, Seurat v3.1.1 was used [PMID:29608179], and global-scaling normalization method was applied with a scale factor of 10,000 and log-transformation of data. This was followed by a scaling linear transformation step, to avoid highly expressed genes having higher weight in downstream analysis.

### Clustering and marker genes

PCA was performed on the scaled data, with a Jackstraw plot to choose how many PCs to retain as an input for Seurat clustering step. All samples were evenly distributed among clusters, except for sample 2V that showed a very distinctive pattern and was grouped in separated clusters. This sample was therefore removed from the expression matrix in further analysis, keeping 3 samples in the Y NMR group (1J, 5J, 6J) and two samples in the MA NMR group (3V, 4V). A second clustering step was performed with the five remaining samples using default parameters, Louvain algorithm as the clustering method and a resolution parameter defining the clusters granularity set to 0.4. Marker genes defining each cluster were found via differential expression testing, with a Wilcoxon rank sum test and a log fold change threshold (logFc) of 1. The found marker genes were first compared to a list of 98 genes of interest for cluster identification, extracted from previous single-cell transcriptomic skin data [21, 22]. Then, we also compared the marker genes to published scRNA-seq transcript or protein expression data documenting skin layer specificity (Supplementary Data File 1). Initial 11 clusters were then defined separately based on a specific set of genes and next grouped in the following metaclusters: basal 1, basal 2, intermediate 1, intermediate 2, corneum and immune cells.

### Trajectory

Single-cell trajectory was constructed using M3Drop [PMID:30590489] and Monocle v2.10.1 [PMID:28825705]. Input genes for Monocle trajectory construction were selected using an unsupervised approach via M3Drop result, which identifies differentially expressed genes based on a Michaelis-Menten function for the relationship between mean expression and dropout rate, the relevant genes being the ones shifting above a fitted curve. The default Monocle workflow was then performed to generate the trajectories, with focus on known markers expression profiles (*Krt14*, *Krt10*, *Lor*) to select the appropriate trajectory orientation. We utilized Monocle 2.6.425 to order cells in pseudotime based on their transcriptomic similarity. Pseudotime-dependant genes have been identified using differential GeneTest function from Monocle. Those genes were the most significant in this context and Monocle reconsidered this list of 820 genes and kept the majority of them for the analyses (791 pseudotime-dependent genes). The differential test was performed on genes used to construct Monocle trajectory on cells from Young samples and selected according to q-value (lower than 0.05). Clustering of these genes was done by Monocle functions. Heatmaps for Y and MA samples used this clustering of genes.

### Enrichment analysis

Using the Cytoscape v3.7.2 software and BiNGO plugin (binomial statistical test and Bonferroni correction, threshold with adjusted p<0.01), we determined which Gene Ontology (GO) categories are statistically overrepresented in a set of genes of a biological network in the newly defined metaclusters on Mus Musculus annotations. For specific analysis, a Fisher exact-test was performed on a list of genes compiled from REACTOME (ROS) and GO terms (aging and wound healing) databases. Results with uncorrected p-value ≤ 0.05 were considered enriched.

### Wound healing procedure

NMR were anesthetized and punch biopsy devices were used to create four 5 mm surgical wounds on the backs of the animals. All tissues above the panniculus carnosus were excised. Wounds were left uncovered until analysis. Wound tissues were harvested at day 7 post injury and stored at -80° C after formalin fixation and sucrose washing/immersion. Standardized images of the wounds were obtained at various time points, with a Sony Cybershot 10.1-megapixel DSC-W180 digital camera (Sony, Tokyo, Japan). The images were used to measure the wound closure rate using ImageJ software every 2-3 days.

### Statistics

The data were analyzed using Chi^2^ statistic test, Wilcoxon test, Unpaired Student’s t-test, Mann–Whitney U test and One-way ANOVA. n indicates the number of independent experiments performed. In all histograms, asterisks correspond to: *p < 0.05, **p < 0.01, ***p < 0.001, ****p < 0.0001.

## Supplementary Material

Supplementary Figures

Supplementary Table 1

Supplementary Data File 1

Supplementary Data File 2

Supplementary Data File 3

Supplementary Data File 4
