# Supplementary material for: Single-cell transcriptomics reveals age-resistant maintenance of cell identities, stem cell compartments and differentiation trajectories in long-lived naked mole-rats skin
Source: Aging (Albany NY). 2022 May 4;14(9):3728–56. doi: 10.18632/aging.204054 (PMC9134947; doi:10.18632/aging.204054)
Supplement: Supplementary Figures [file aging-14-204054-s001.pdf]

SUPPLEMENTARY FIGURES

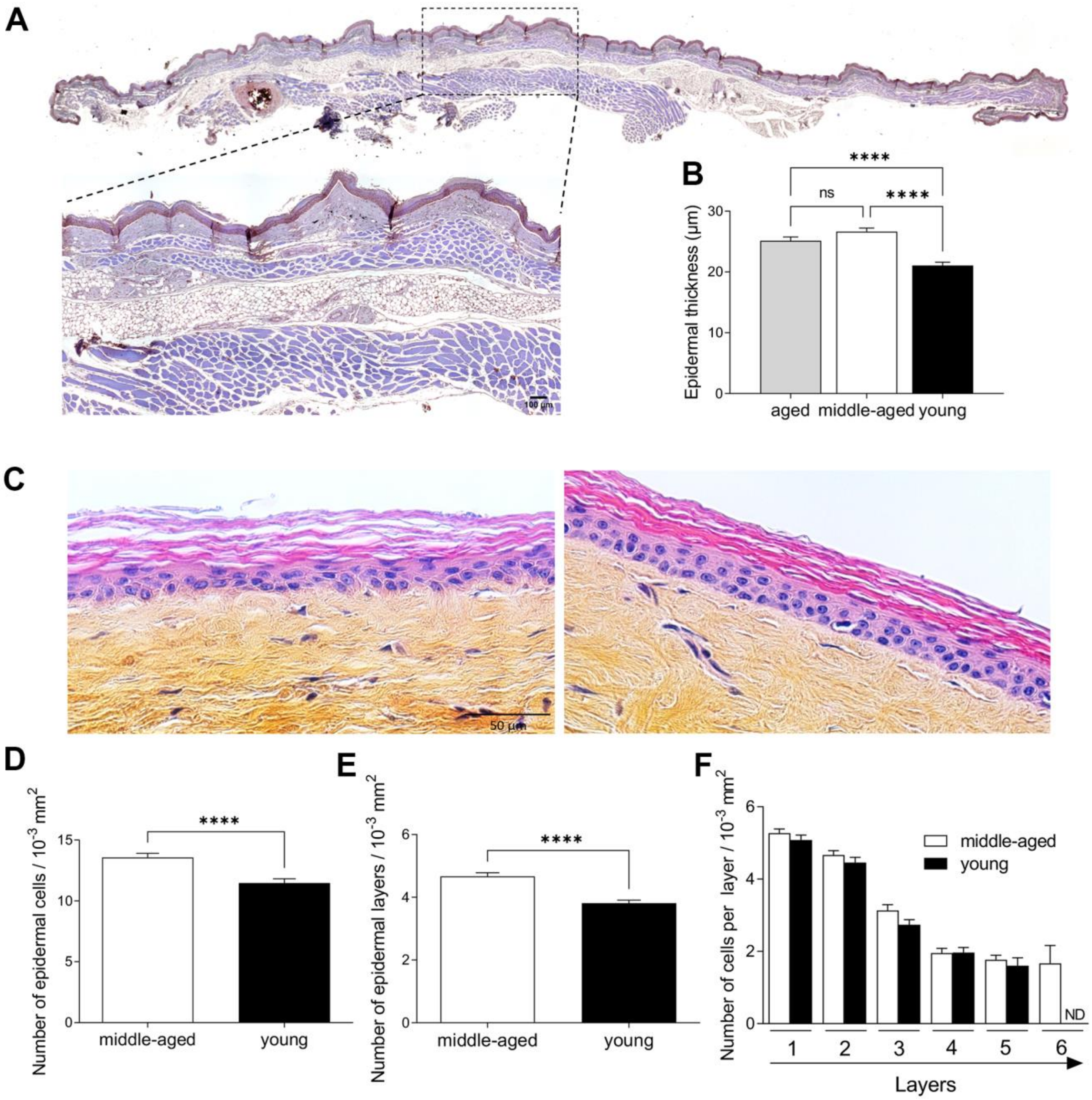

Supplementary Figure 1. Epidermal morphology of young, middle-aged and aged naked mole-rat (NMR). (**A**) HE staining with a higher magnification of an aged (25 years-old) NMR skin. Scale bar = 100 µm. (**B**) Histogram shows the increased epidermal thickness of aged and middle-aged as compared to young NMR back skin. (**C**) Examples of two aged (20 years-old) NMR skin histology using HES staining. Scale bar = 50 µm. (**D**) Histogram showing the number of epidermal cells in middle-aged versus young NMR per surface. (**E**) Histogram showing the number of epidermal layers in middle-aged vs young NMR. (**F**) Histogram showing cell number per layer in middle-aged versus young NMR, per surface. An additional external layer was found in middle-aged samples. n=4 animals per group except for the aged animals (n=3). Bars: SEM. *represents differences between the age groups.****p < 0.0001 in ANOVA and Student's t-test. ND : non detectable. ns = non significant.

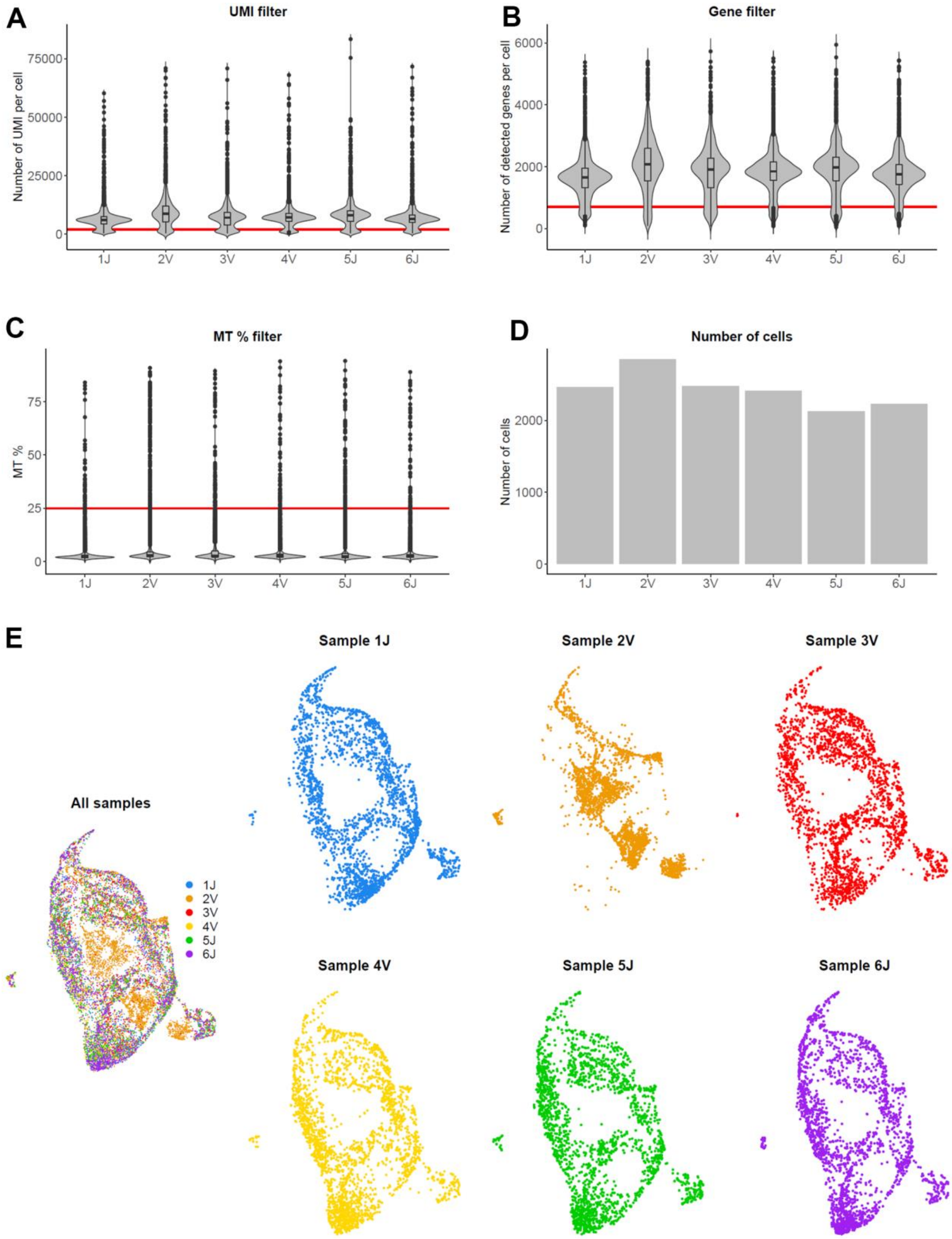

Supplementary Figure 2. Quality controls of scRNA-seq. (**A**) Violin plot of the number of UMIs per cell for each animal. A minimum threshold is applied at 2,000 (red line). (**B**) Violin plot of the number of genes detected per cell for each animal. A minimum threshold is applied at 700 (red line). (**C**) Violin plot of the percentage of mitochondrial reads per cell for each animal. A minimum threshold is applied at 25% (red line). (**D**) Bar plot of the number of cells for each animal. (**E**) Uniform Manifold Approximation and Projection (UMAP) representation for all samples grouped and individualized, colored by individual, including the outlier (2V) removed in the rest of the study.

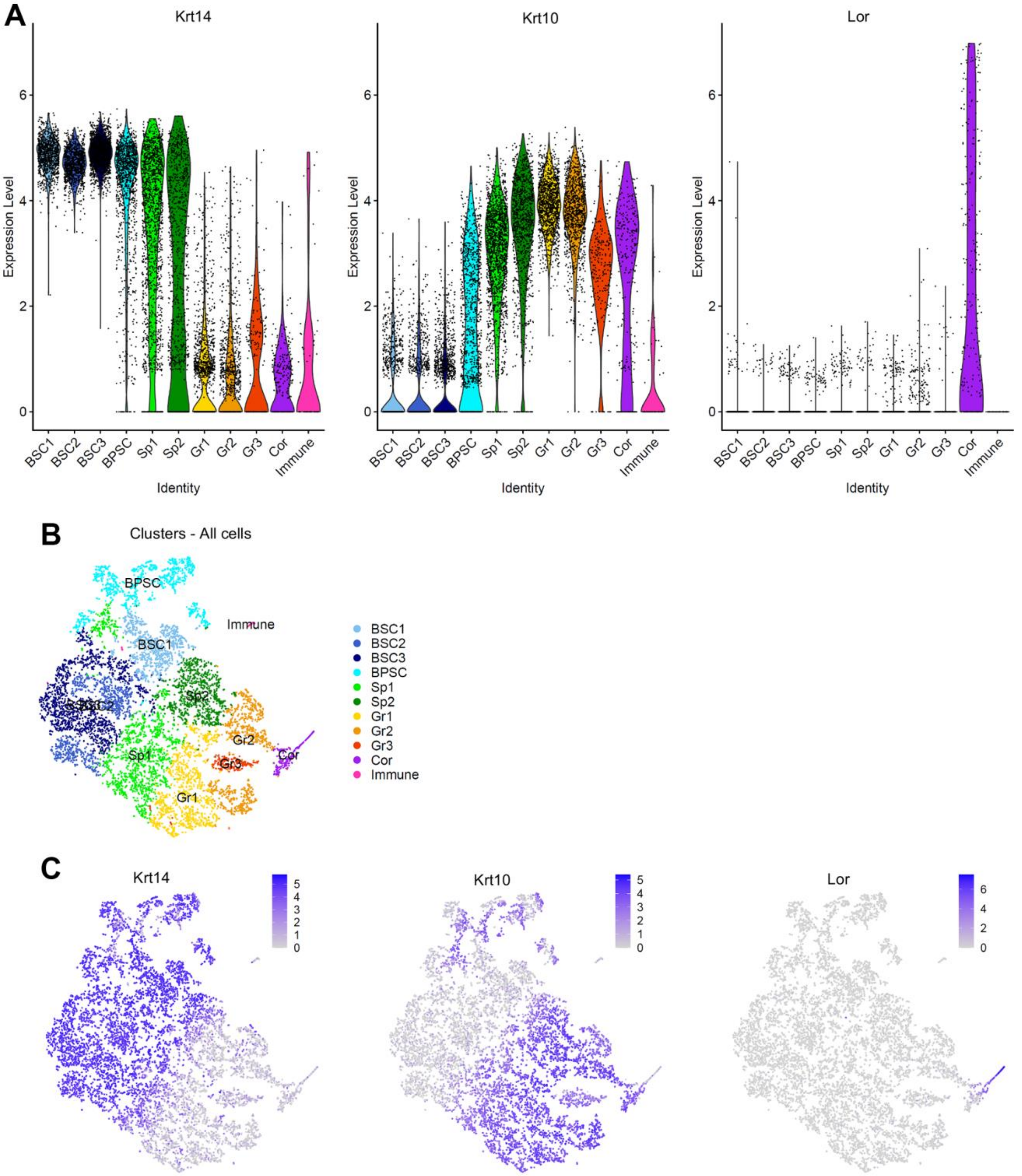

Supplementary Figure 3. scRNA-seq of NMR epidermal cells define 3 cellular states. (**A**) Violin plots of *Krt14*, *Krt10* and *Lor* marker genes expressed by NMR epidermal cells, leading us to confirm 3 cellular states of keratinization. (**B**) t-distributed Stochastic Neighbor Embedding (t-SNE) visualization of epidermal cells clustering. (**C**) t-SNE visualization of *Krt14, Krt10 and Lor* marker genes expressed by NMR epidermal cells. BSC = basal and stem cells; BPSC = basal proliferating and stem cells; Sp = Spinous layer cells; Gr = granular layer cells; Cor = corneous layer cells.

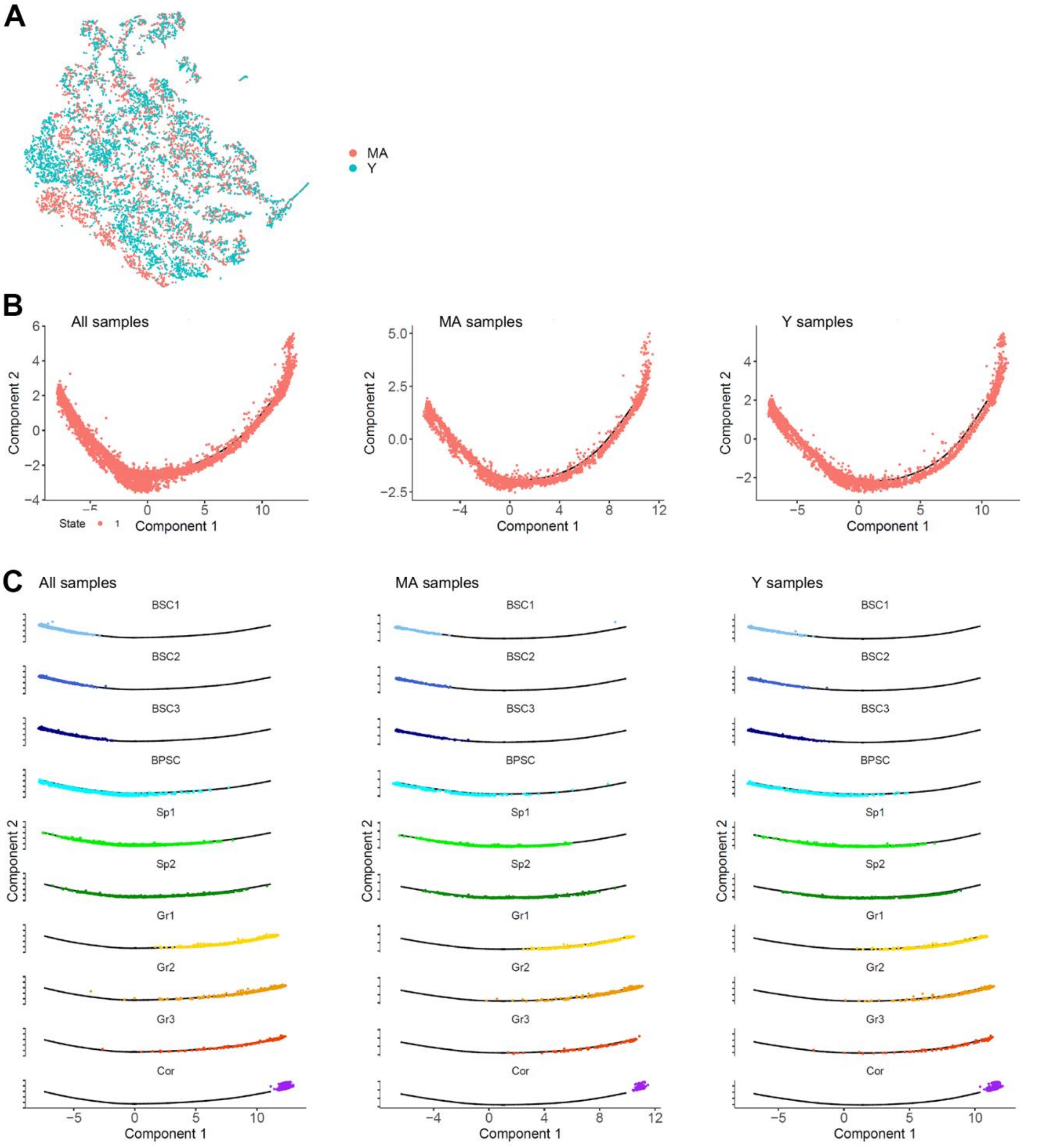

Supplementary Figure 4. scRNA-seq of young versus middle-aged NMR epidermis. (**A**) t-distributed Stochastic Neighbor Embedding (t-SNE) visualization of epidermal cells where both middle-aged (MA, red) and young (Y, blue) NMR were jointly projected on the same plot, showing an overlap of the 2 age groups tested. (**B**) Unsupervised differentiation trajectories for merged, MA and Y keratinocytes colored by Monocle state of differentiation. A single and similar trajectory in both samples was found. (**C**) Unsupervised differentiation trajectories for merged, MA and Y keratinocytes segregated by cluster. No difference between the 2 age groups was found. BSC = basal and stem cells; BPSC = basal proliferating and stem cells; Sp = Spinous layer cells; Gr = granular layer cells; Cor = corneous layer cells.

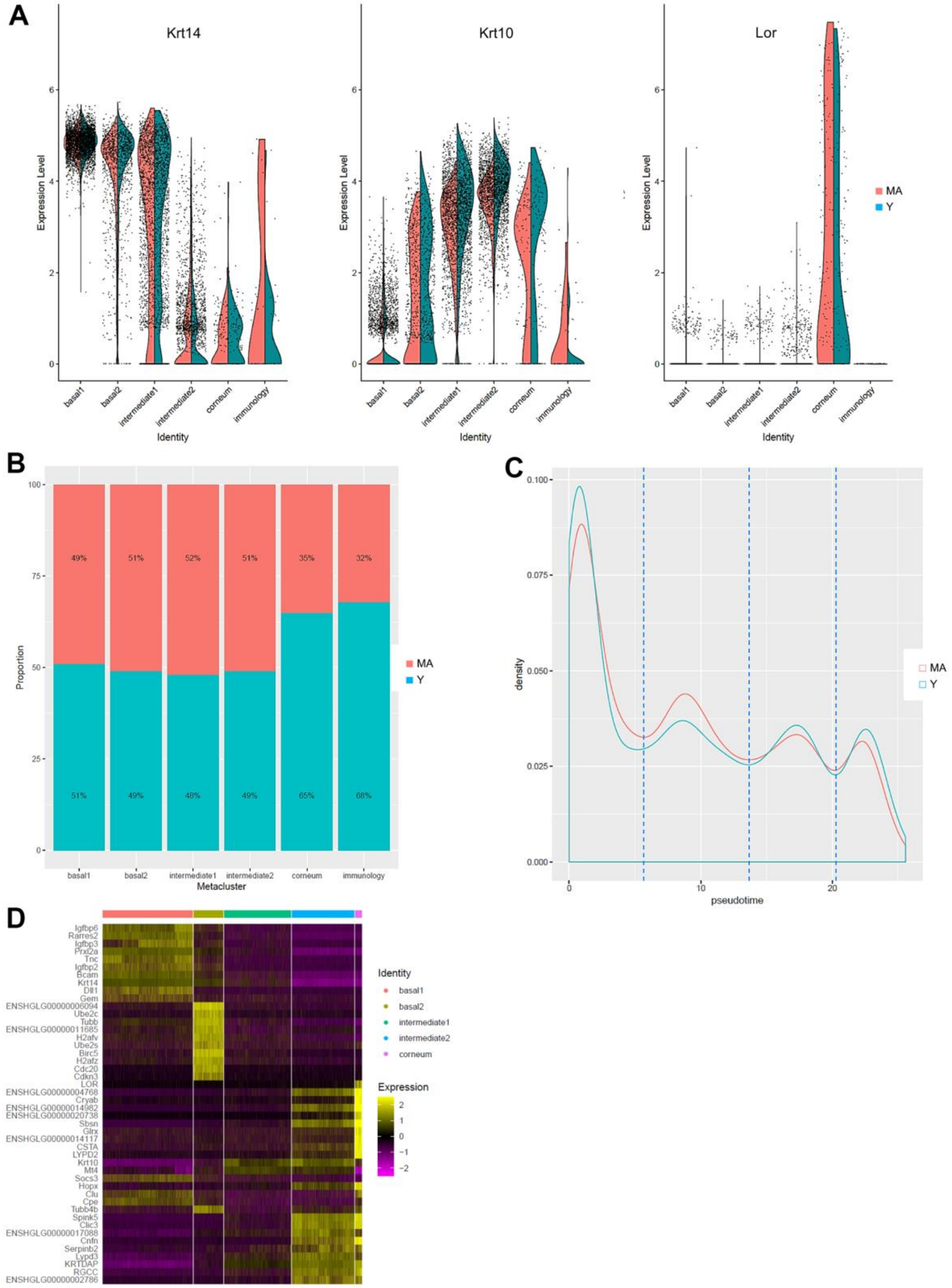

Supplementary Figure 5. Metaclusters identification and pseudotime estimation and of young and middle-aged NMR epidermal cells. (**A**) The expression of *Krt14, Krt10 and Lor* marker genes visualized with violin plots confirmed the cellular states of the newly defined metaclusters for middle-aged (MA, red) and young (Y, blue) NMR. (**B**) Bar graph representing the relative proportion of epidermal cells in each metacluster between MA (red) and Y (blue) animals. No significant difference between the 2 age groups was noted (Chi2 statistic test). (**C**) Distributions plotting the frequency of MA and Y cells as a function of the pseudotime on its scale. Distributions were similar between the 2 age groups. (**D**) Heatmap of most differentially expressed genes for each newly defined metacluster. Cells are represented in columns, and genes in rows.

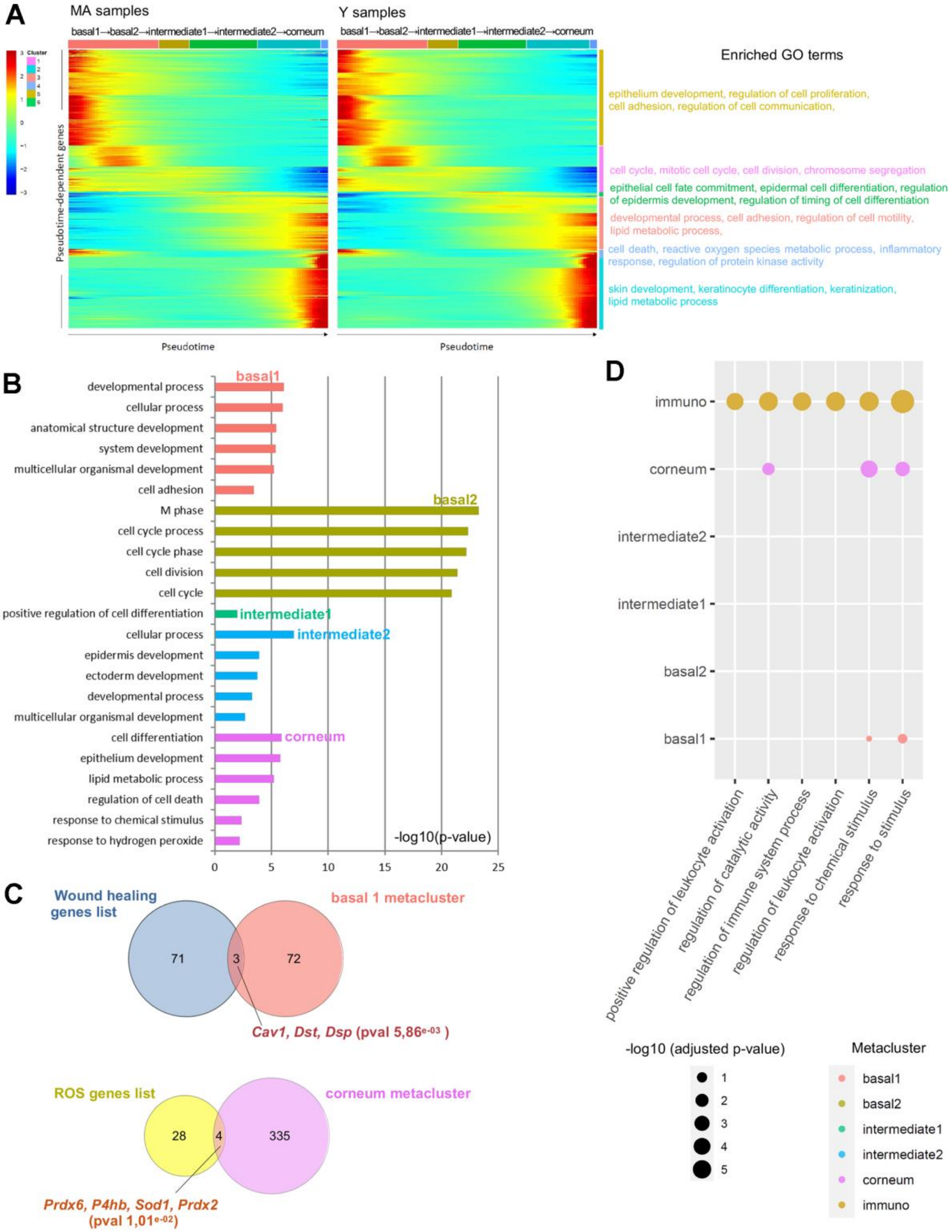

Supplementary Figure 6. Metaclusters characterization of NMR keratinocytes. (**A**) Heatmap pf pseudotemporal dynamics of the pseudotime-dependent genes in MA (middle-aged) and Y (young) NMR (left panel). Each row (i.e., gene) is normalized to its peak value along the pseudotime. 6 distinct clusters or stages during pseudotime are represented by colored bars on the side. The most significant enriched GO terms in each gene cluster are listed (right panel). The gene ordering is strictly the same between MA and Y heatmaps. (**B**) Barplot representing a selection of the most significant enriched Gene Ontology (GO) terms (from BiNGO analysis) in each keratinocytes metaclusters (except immune cluster) sorted by p-value. Barplot represents the adjusted p-value. (**C**) Venn diagram depicting the genes overlap significantly overrepresented in basal 1 ((pval 5,86e-03) and corneum metaclusters (pval 1,01e-02) as compared respectively with wound healing and ROS gene lists. (**D**) Bubble plot representing the immune Gene Ontology (GO) terms released by the BiNGO analysis. The bubbles represent the adjusted p-value of these terms in the immune cluster and in the other clusters when they emerged.

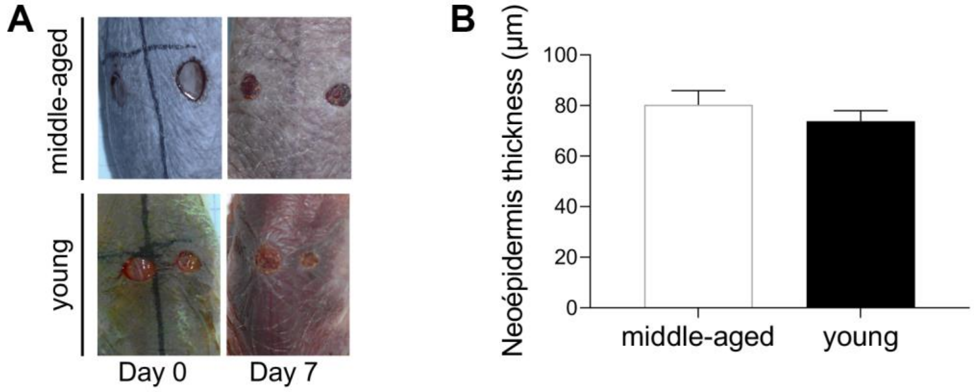

Supplementary Figure 7. Wound healing of young versus middle-aged NMR. (**A**) Photomicrographs of back skin wounds for middle-aged and young NMR at day 0 and day 7 post wounding. Skin wounds were harvested at day 7 to analyze re-epithelialization. (**B**) Histogram showing the neoepidermal thickness (µm) of middle-aged and young NMR wounded back skin at day 7. Bars: SEM. A Mann–Whitney U test was used. n=2 animals per group.
