# Supplementary material for: Single-cell transcriptomics reveals age-resistant maintenance of cell identities, stem cell compartments and differentiation trajectories in long-lived naked mole-rats skin
Source: Aging (Albany NY). 2022 May 4;14(9):3728–56. doi: 10.18632/aging.204054 (PMC9134947; doi:10.18632/aging.204054)
Supplement: Supplementary Table 1 [file aging-14-204054-s002.pdf]

SUPPLEMENTARY TABLE

Supplementary Table 1. Characteristics of antibodies used for immunohistochemistry.

Reagent or resource	Dilution	Species	Source	Identifier	Section method
**Antibodies**					
Keratin-14	1:1000	rabbit	Biolegend	905301	paraffin
Keratin-10	1:1000	rabbit	Biolegend	905401	cryo
Loricrin	1:1000	rabbit	Biolegend	905101	paraffin
Filaggrin	1:500	rabbit	Biolegend	905801	paraffin
Laminin-5	1:200	rabbit	Abcam	ab14509	cryo
ITGB4	1:250	rabbit	Abcam	ab182120	paraffin
Ki67	1:400	rabbit	Abcam	ab15580	cryo
Collagen I	1:250	rabbit	Abcam	ab21286	paraffin
p16	1:100	rabbit	ThermoScientific	PA1-30670	cryo
Hyaluronic acid	1,6:100	bovine	Calbiochem	385911	paraffin
IGFBP3	1:500	rabbit	Abcam	272857	cryo
